# Cytokines and Chemokines in Irritant Contact Dermatitis

**DOI:** 10.1155/2013/916497

**Published:** 2013-11-25

**Authors:** Haur Yueh Lee, Marco Stieger, Nikhil Yawalkar, Masato Kakeda

**Affiliations:** ^1^Department of Dermatology, Inselspital, University of Bern, 3010 Bern, Switzerland; ^2^Department of Dermatology, Mie University Graduate School of Medicine, Tsu, Mie 514-8507, Japan

## Abstract

Irritant contact dermatitis is a result of activated innate immune response to various external stimuli and consists of complex interplay which involves skin barrier disruption, cellular changes, and release of proinflammatory mediators. In this review, we will focus on key cytokines and chemokines involved in the pathogenesis of irritant contact dermatitis and also contrast the differences between allergic contact dermatitis and irritant contact dermatitis.

## 1. Introduction

Irritant contact dermatitis (ICD) is an inflammatory response of the skin to various external stimuli. It arises as a result of activated innate immunity to direct injury of the skin without prior sensitization [1–3]. ICD is a complex reaction modulated by both intrinsic and extrinsic factors [2–4]. Intrinsic factors which influence the susceptibility to ICD include genetic predisposition, for example, atopic diathesis, age, sex, and body region. Extrinsic factors include the inherent nature of the irritants, exposure volume, concentration, duration, repetition, and the presence of further environmental and mechanical factors.

ICD has a spectrum of clinical features which can be divided into several different categories depending on the irritant and its exposure pattern. Ten clinical subtypes have been proposed [2]. The influence of irritants on various cytokines/chemokines has not been well delineated so far, although it is plausible that different environmental insults and the subsequent variation in cytokines/chemokines expression could result in distinct clinical phenotypes.

In this review, we discuss the pathophysiological mechanisms involved in ICD with a focus on key cytokines and chemokines as well as their cellular source in the skin. Furthermore, we highlight the key differences between ICD and allergic contact dermatitis (ACD).

## 2. Pathophysiology of Irritant Contact Dermatitis

Previously thought of as an immunologic inert process, at present there is increasing evidence showing that ICD is a complex, interlinked process involving perturbations in the skin barrier integrity, cellular changes, and release of various proinflammatory mediators [5, 6].

### 2.1. Irritants and Skin Barrier Integrity

Integrity of the epidermal barrier function plays an important role in the interaction and the response of the human skin to irritants [7]. Patients with atopic dermatitis are known to have an epidermal barrier dysfunction and have an augmented response to various exogenous irritants [8]. In particular, atopic dermatitis and filaggrin null alleles are associated with an increased susceptibility and severity to ICD [9, 10]. Recently, it has been shown that filaggrin loss-of-function mutation is associated with an enhanced expression of IL-1, which plays a central role in the initiation of ICD [11].

The mechanisms leading to damage of the skin barrier are also dependent on the intrinsic nature of the irritant. Organic solvents such as acetone can extract lipids from the stratum corneum, thereby leading to disruption of the epidermal barrier [12]. Anionic surfactants like sodium lauryl sulphate (SLS) can damage protein structures such as keratin, involucrin, profilaggrin, and loricrin, exposing new water binding sites and causing hyperhydration of the stratum corneum and disorganization of the lipid bilayers [13–16]. The end result of this damage to the skin barrier is the activation of the innate immunity with its cellular changes and production of proinflammatory cytokines, such as IL-1*α*. Simultaneously, barrier disruption also induces reparative processes to restore homeostasis [17]. 

### 2.2. Key Cellular Components and Mediators in ICD

#### 2.2.1. Keratinocytes

Keratinocytes play a major role in the production of immune mediators in ICD. The disruption of the skin barrier leads to release of preformed IL-1*α* [18], which represents an initial step in the inflammatory cascade of ICD. Numerous *in vitro* studies have also shown that various irritants induce IL-1*α* expression in keratinocytes [5, 6, 19–22]. Activation of IL-1*α* is subsequently thought to stimulate further production of proinflammatory cytokines and chemokines such as IL-1*β*, TNF-*α*, IL-6, and CXCL8 (IL-8) by surrounding epidermal and dermal cells [23, 24].

Unlike IL-1*α*, which is constitutively produced, IL-1*β* is secreted as a biologically inactive precursor that is cleaved into an active 17.5 kDa molecule by a protease not normally present in resting keratinocytes [25]. IL-1*β*-converting enzyme (ICE) is a unique processing enzyme involved in the production of active IL-1*β*. In activated keratinocytes, ICE has been readily detected following incubation with irritants such as phorbol myristate acetate (PMA) or SLS [26], indicating that activation of ICE may represent a key pathogenic step in ICD elicited through certain irritants. 

Together with IL-1*α*, the subsequent actions of IL-1*β* are pleiotropic and involve activation of dendritic cells and T cells, further cytokine and chemokine production, and upregulation of adhesion molecules such as ICAM-1 on endothelial cells and fibroblasts [5, 6, 24], which can all lead to perpetuation of cutaneous inflammation.

Another key cytokine in ICD is TNF-*α*. Upregulation of TNF-*α* in the skin has been reported following application of irritants such as dimethyl sulfoxide, PMA, formaldehyde, tributyltin, and SLS [20, 27–29]. Moreover, the importance of TNF-*α* in ICD has previously been demonstrated in irritant reactions which were inhibited *in vivo* by administration of antibodies to TNF [30]. The effects of TNF-*α* are also pleiotropic, leading to increased expression of major histocompatibility complex class II molecules, upregulation of cell adhesion molecules such as ICAM-1 on keratinocyte and endothelial cells [31, 32], and further induction of inflammatory mediators such as IL-1, IL-6, GM-CSF, IFN-*γ*, and CXCL8 [23, 33]. In addition, TNF-*α* in concert with IL-1*α* particularly acts as primary alarm signals, which triggers the release of secondary CCL20 (Macrophage Inflammatory Protein-3) and CXCL8 chemokine signals [34, 35]. These increased levels of CCL20 and CXCL8 have the potential to initiate infiltration of immune cells such as CCR6+ T cells and immature dendritic cells into an area of the skin that is exposed to the irritant [36].

Further support for a central role of IL-1*α* and TNF-*α* in the pathogenesis of ICD include recent studies which have shown that certain genetic polymorphisms are associated with increased or decreased risk of developing ICD. Individuals with TNFA-238 polymorphisms have a reduced risk of developing ICD whereas individuals with TNFA-308 alleles have an increased risk of ICD [37]. Similarly, individuals with IL1A-889 C/T polymorphisms are associated with a protective effect to the development of ICD [38]. 

Other cytokines and factors that have been implicated in the pathogenesis of ICD and which are also produced by keratinocytes include vascular endothelial growth factor (VEGF) [21, 39, 40] and IL-6 [19, 27, 39]. VEGF which is mainly secreted by keratinocytes is a potent mediator of angiogenesis that stimulates the migration and proliferation of endothelial cells, facilitates vascular permeability, and induces the expression of adhesion molecules ICAM-1 and VCAM-1 on endothelial cells [39]. IL-6,which is upregulated by various irritants, induces infiltration of mononuclear cell and is believed to play an important role in perpetuating skin inflammation. However, a recent study has shown that IL-6 may also exert some anti-inflammatory effects in ICD and that these effects may be dependent on the chemical nature of the irritant [41]. Furthermore, counterregulatory cytokines such as IL-10 [27, 42] and IL-1 receptor antagonists [21] especially in repeated irritant application are also produced to limit inflammation.

#### 2.2.2. Fibroblasts

Dermal involvement is common in ICD due to either penetration of the irritant chemical to the dermis or indirectly through mediators derived from keratinocytes. Fibroblasts have been associated with maintaining homeostasis of the skin immune system by their interactions with the keratinocytes. The release of keratinocytes derived IL-1*α* activates fibroblasts to release other active mediators such as CXCL8, CXCL1 (GRO-*α*), and CCL2 (monocyte chemotactic protein-1/MCP-1) [43]. In addition, TNF-*α* dependent secretion of CCL2 and CCL5 (RANTES) plays a role in initiating migration of irritant-exposed human Langerhans cells (LCs) out of the epidermis [44–46].

#### 2.2.3. Endothelial Cells

Following exposures to irritants, there is an upregulation of adhesion molecules and chemokines on endothelial cells which can facilitate the migration of further immune cells like dendritic cells, macrophages, and T cells into the skin. Interestingly, CCL21 has been reported to be upregulated on dermal lymphatic endothelial cells in ICD [47]. This is thought to facilitate the emigration of CCR7+ dendritic cells (DCs) from the skin.

#### 2.2.4. Dendritic Cells

The role of DCs and their cytokines in ICD is not well characterized. Epidermal LCs have been shown to migrate into the dermis after topical exposure of irritants to the skin, despite the supposed independence of ICD from adaptive immunity [44, 48]. This migration is likely to occur due to the upregulation of IL-1 and TNF-*α* by irritants. Furthermore, migration to the dermis occurs under the influence of CCL2 (MCP-1) and CCL5 (RANTES), which are secreted by fibroblasts [44]. In addition, it has been shown that there is an IL-10 dependent postmigration phenotypic switch from CD1a+ LCs into CD14+CD68+ macrophage-like cells in ICD [46]. The significance of this migration and phenotypic switch is unclear although it is postulated that this is an important escape mechanism to protect LCs from cell death by harmful toxic agents. These transformed CD14+CD68+ macrophages may also have a role in rapidly removing damaged tissue as a result of skin barrier disruption, and lastly this phenotypic switch may also serve to maintain immunological ignorance, thereby avoiding the generation of collateral autoimmunity [46].

#### 2.2.5. Lymphocytes

The role of skin infiltrating T lymphocytes in ICD is also not well defined. In acute reactions of ICD, cellular infiltrates consisting of mainly CD4+ lymphocytes are seen with an increased level of IL-2 and IFN-*γ* (Th1-associated cytokines) as well as CD8+ cytotoxic T cells [20, 49]. However, it has been shown that Th1 associated CXCR3 ligands such as CXCL9, 10, and 11 are among the most differentially expressed chemokines discriminating between ICD and ACD [50]. These chemokines were expressed at significantly lower levels in ICD compared to ACD. Further studies would be needed to clarify the role and subsets of T lymphocytes involved. Recently, Th17 cells which are novel subset of CD4+ T cells have been shown to be implicated in the pathogenesis of ACD. These T-cell subsets induce chemokine and cytokine release from keratinocytes and intensify the ICAM-1 dependent keratinocytes T-cell interaction thus promoting nonspecific T-cell-induced apoptosis. At present, it remains unclear if a similar mechanism exists for ICD [51–53].

### 2.3. Summary of Cytokines/Chemokines Activation Cascade in ICD

**Figure 1:**
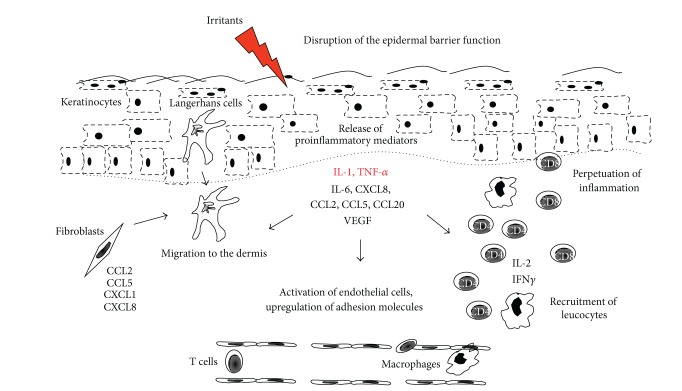
Immunological mechanisms in irritant contact dermatitis. Following irritant insult, there is disruption of the epidermal barrier with release of proinflammatory mediators, that is, IL-1 and TNF-*α*. This results in stimulation of further cytokine and chemokines production, for example, by keratinocytes, fibroblasts, and endothelial cells, upregulation of adhesion molecules on endothelial cells, and the subsequent recruitment of leucocytes thereby perpetuating ongoing inflammation.

Although the precise cytokines/chemokines activation cascade in ICD is still unclear, it is likely that the primary cytokines involved following irritant exposure are IL-1 and TNF-*α*. The synergistic effects of these two cytokines result in the further activation and release of secondary cytokines/chemokines such as IL-2, IL-6, GM-CSF, IFN-*γ*, VEGF, CXCL8, CCL2, CCL5, and CCL20 and expression of cell adhesion molecules [4, 5, 23, 30, 31]. A postulated model is shown in Figure 1. The various cytokines/chemokines and mediators involved in ICD are also summarized in Table 1. The myriad of cytokines and cell types involved in ICD demonstrates that the complexity of the skin response to irritants and interindividual variations in the level of cytokines present or produced in the skin is likely responsible for the nature of irritants and intensity of the irritation reaction.

## 3. Comparison between ACD and ICD

Despite some distinct pathological differences, many common features such as certain histopathological (e.g., cellular infiltrate, vasodilatation) and molecular (e.g., cytokines/chemokines production, upregulation of endothelial adhesion molecules) alterations exist between ICD and ACD [54, 55]. Such similarities have also been attributed to the irritant potential of allergens which strongly contributes to their allergenicity [56]. In the early phases, it is likely that IL-1 and TNF-*α* driven innate immune responses are involved in both ICD and ACD. In later phases of ICD, skin inflammation is still critically dependent on innate responses. However, in ACD adaptive immune responses involving antigen-specific T cells take over to amplify skin inflammation [50]. In recent years, some molecular differences between ICD and ACD have been identified. In particular, CXCR4 and CCR7 expressions on LCs have been shown to be upregulated after allergen but not by irritant exposure [46]. CXCR4 and CCR7 are important chemokine receptors which facilitate allergen-induced LC migration toward the lymph vessels via a two-step CXCR4-CXCL12 and CCR7-CCL19/CCL21 interaction [57]. Moreover, the expression of CXCL9, CXCL10, and CXCL11 has been shown to be specifically upregulated in ACD [50]. In addition, *in vitro* studies using monocyte-derived DCs have shown that certain phenotypic alterations of immature DCs such as upregulation of surface expression markers (CD54, CD86, and HLA-DR) as well as production of IL-1*β* [58] and CXCL8 [59] are increased in ACD compared to ICD. Previous studies involving gene expression analysis have also demonstrated that allergens but not irritants may lead to upregulation of certain genes such as CCL23, CCL4, CYP27A1, HML2, NOTCH3, S100A4, and SLAM in DCs, thus providing the basis for approaches to identify skin-sensitizing chemicals [60].

## 4. Conclusion

Although the precise pathomechanisms of ICD still remain to be elucidated, there is increasing evidence that a myriad of cytokines and chemokines as well as immune cells are actively involved in ICD. Greater understanding of these mechanisms and differences between ACD and ICD will aid in the evaluation of irritants and assessment of skin damage as well as therapeutics. 

## Figures and Tables

**Table 1:** Key cytokines and chemokines involved in ICD.

Cytokine	Source	Function
Interleukin-1	Keratinocytes Langerhans cells/dendritic cells Monocytes/macrophages T cells Endothelial cells	Proinflammatory Chemoattractant for T and B cells Upregulates adhesion molecule Induces IL-1, IL-2, IL-4, IL-6, IFN-*γ*, CXCL8, and CCL20 Aids Langerhans cell migration

Interleukin-6	Keratinocytes Langerhans cells/dendritic cells Monocytes/macrophages Fibroblasts Endothelial cells	Proinflammatory Chemotactic for neutrophils and T cells Keratinocyte proliferation

Interleukin-8 (CXCL8)	Keratinocytes Monocytes/macrophages Fibroblasts Neutrophils T cells Endothelial cells Lymphocytes	Proinflammatory Chemotaxis Activation of neutrophils Basophil release of histamine

Interleukin-10	Keratinocytes T cells	Anti-inflammatory Inhibits production of IL-1*α*, IL-1*β*, IL-2, IL-3, IL-6, IL-8, TNF-*α*, MIP-1*α*, IFN-*γ*, M-CSF, and GM-CSF Downregulates MHC class II Downregulates adhesion molecules

GM-CSF	Keratinocytes Melanocytes T cells Endothelial cells Mast cells	Proinflammatory Enhances effector function of monocytes and neutrophils

IFN-*γ*	Lymphocytes Keratinocytes	Proinflammatory Induces/enhances MHC class II Upregulates cellular adhesion molecules

TNF-*α*	Keratinocytes Dendritic cells Monocytes/macrophages Mast cells Fibroblasts Lymphocytes	Proinflammatory Activates T cells, macrophages, and granulocytes Upregulates MHC classes I and II Induces IL-1, IL-6, IL-8, TNF, GM-CSF, M-CSF, G-CSF, PDGF, and VEGF Cellular adhesion molecule expression

VEGF	Keratinocytes	Proinflammatory Induces endothelial cell permeability Promotes angiogenesis Increases expression of adhesion molecules Promotes monocyte migration

CCL2 (MCP-1)	Monocytes/macrophages Dendritic cells Fibroblasts	Chemotactic for monocytes, T cells, and dendritic cells

CCL5 (RANTES)	Keratinocytes Dendritic cells Fibroblasts Mast cells	Chemotactic for T cells, eosinophils, and basophils

CCL20 (MIP-3)	Keratinocytes Lymphocytes Fibroblasts Monocytes	Chemotactic for dendritic cells, lymphocytes, and neutrophils

Adapted and modified from Smith et al. [5].
